# A20 promotes melanoma progression via the activation of Akt pathway

**DOI:** 10.1038/s41419-020-03001-y

**Published:** 2020-09-23

**Authors:** Jinyuan Ma, Huina Wang, Sen Guo, Xiuli Yi, Tao Zhao, Yu Liu, Qiong Shi, Tianwen Gao, Chunying Li, Weinan Guo

**Affiliations:** Department of Dermatology, Xijing Hospital, Fourth Military Medical University, No 127 of West Changle Road, 710032 Xi’an, Shaanxi China

**Keywords:** Melanoma, Oncogenesis

## Abstract

Melanoma is the most life-threatening skin cancer with increasing incidence around the world. Although recent advances in targeted therapy and immunotherapy have brought revolutionary progress of the treatment outcome, the survival of patients with advanced melanoma remains unoptimistic, and metastatic melanoma is still an incurable disease. Therefore, to further understand the mechanism underlying melanoma pathogenesis could be helpful for developing novel therapeutic strategy. A20 is a crucial ubiquitin-editing enzyme implicated immunity regulation, inflammatory responses and cancer pathogenesis. Herein, we report that A20 played an oncogenic role in melanoma. We first found that the expression of A20 was significantly up-regulated in melanoma cell lines. Then, we showed that knockdown of A20 suppressed melanoma cell proliferation in vitro and melanoma growth in vivo through the regulation of cell-cycle progression. Moreover, A20 could potentiate the invasive and migratory capacities of melanoma cell in vitro and melanoma metastasis in vivo by promoting epithelial–mesenchymal transition (EMT). Mechanistically, we found that Akt activation mediated the oncogenic effect of A20 on melanoma development, with the involvement of glycolysis. What’s more, the up-regulation of A20 conferred the acquired resistance to Vemurafenib in *BRAF*-mutant melanoma. Taken together, we demonstrated that up-regulated A20 promoted melanoma progression via the activation of Akt pathway, and that A20 could be exploited as a potential therapeutic target for melanoma treatment.

## Introduction

Melanoma is the most lethal skin cancer that is the malignant transformation from epidermal melanocytes. In 2018, there are estimated 96,480 new cases of melanoma emerging and over 7230 deaths among them in the United States^1^. Despite the progressive elucidation of melanoma pathogenesis and the revolutionary advances of targeted therapy and immunotherapy^2^, the prognosis for patients remains unoptimistic, especially for those with metastasis and resistance to current therapies^3^. Therefore, to further investigate the mechanism underlying melanoma pathogenesis and treatment resistance could be helpful for melanoma patients.

Inflammation-related signals, particularly pro-inflammatory cytokines like tumor necrosis factor α (TNFα), have been proved essential for maintaining cell survival in melanoma^4^. A20, which is encoded by *Tumor Necrosis Factor α- Induced Protein* 3 (*TNFAIP3*), was initially identified as a primary responsive gene induced by TNF-α^5^. It was a crucial negative regulator of immunity and inflammatory response via the suppression on NF-κB^6^. Of note, the loss-of-function of A20 could trigger inflammatory response and contribute to the onset and development of many autoimmune diseases^6^. Apart from the role in immunity, A20 is also highly related to tumor biology. Up-regulated A20 acted as an oncogene in breast cancer to facilitate epithelial–mesenchymal transition and tumor metastasis via the ubiquitination of Snail1^7^. However, the loss of A20 contributed to the progression of MYD88_L265P_-driven non-Hodgkin lymphoma^8^. In addition, the down-regulation of A20 by its upstream regulator miR-19a promoted the development of colitis-associated colon cancer^7,9^. These reports indicate that while A20 is a potential therapeutic target for cancer, its effect is tumor type-specific. For melanoma, a previous study revealed that A20 mediated the effect of IL-17RC on the progression of xenograft tumor of B16 melanoma cells^10^. In addition, the suppression of A20 in myeloid-derived suppressor cells or dendritic cells could potentiate the anti-tumor immunity and restrain the progression of implanted melanoma in mice^11,12^. These reports have partially elucidated the biological role of A20 in melanoma, focusing on anti-tumor immunity. However, the expression status of A20 in human melanoma cell and its influence on melanoma pathogenesis, especially on the malignant behavior of melanoma cell, yet to be fully elucidated.

Akt is a serine-threonine kinase partially activated through the phosphorylation of Thr-308 and fully activated upon Ser-473 phosphorylation^13^. Notably, Akt is constitutively activated in up to 70% of human melanomas and plays an important role in melanoma pathogenesis^14^. Increased phosphorylation of Akt was reported to be highly correlated with the clinical stages and the thickness of melanomas, as well as the prognosis of patients^15^. Moreover, Akt could simultaneously stabilize cells with extensive mitochondrial DNA mutation and promote the expression of the ROS-generating enzyme NOX4 to increase angiogenesis and superoxide generation, fostering more aggressive tumor behavior in melanoma^16^. The oncogenic effect of Akt could be possibly attributed to its multiple biological functions, among which the activation of glycolysis might provide sufficient energy and intermediate metabolite for the rapid proliferation of tumor cells^17^. However, the upstream regulatory mechanism of Akt activation and the concomitant glycolysis in melanoma remains elusive.

In the present study, we first found that A20 expression was remarkably up-regulated in melanoma cells. Subsequently, the function of A20 in melanoma growth and metastasis was examined both in vitro and in vivo. Furthermore, the mechanism underlying the oncogenic role of A20 was investigated, involving the regulation of Akt and glycolysis. Ultimately, we examined the role of A20 in the acquired resistance to Vemurafenib in *BRAF*-mutant melanoma.

## Results

### A20 expression is significantly up-regulated in melanoma

**Fig. 1:**
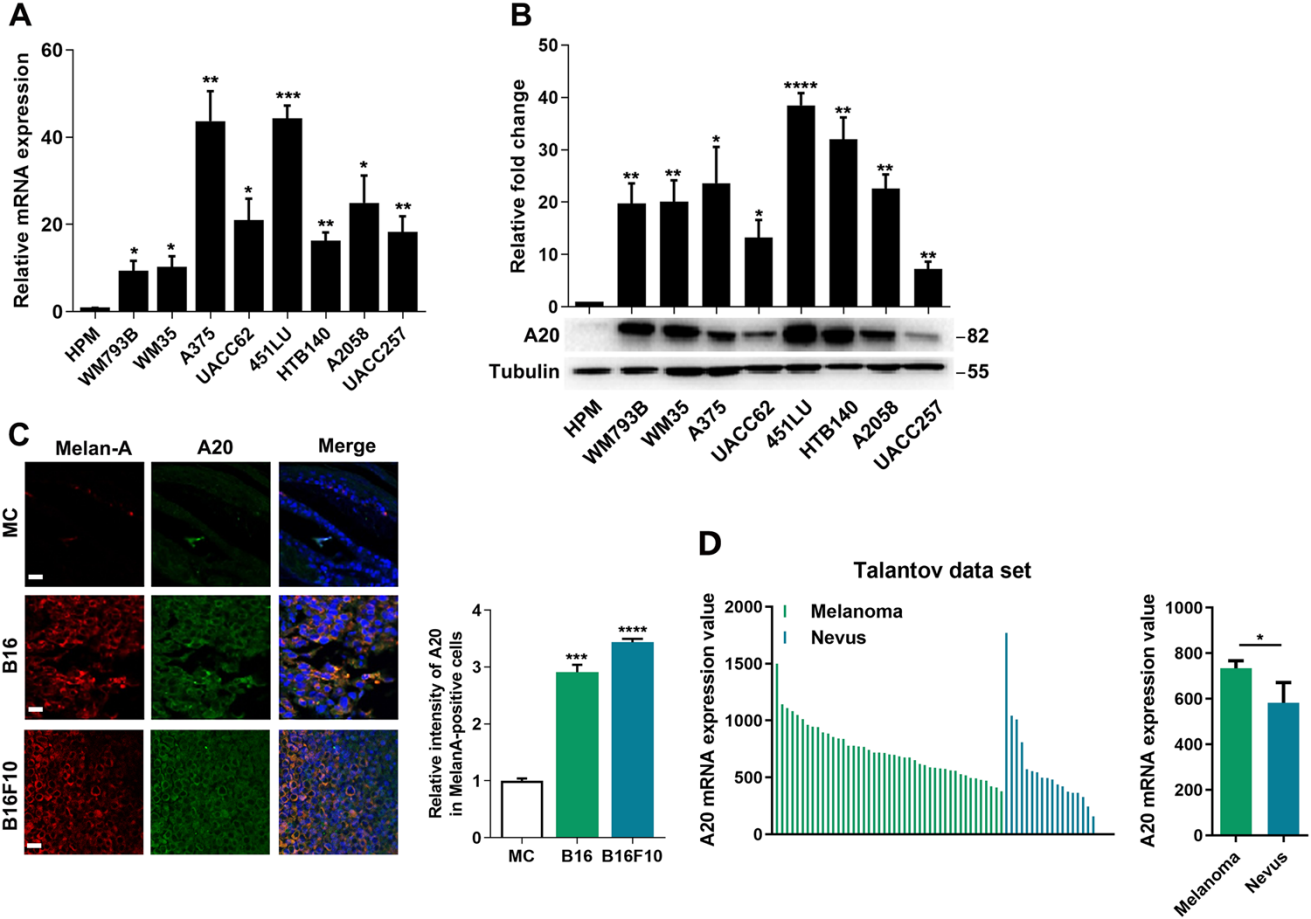
A20 is significantly up-regulated in melanoma. **a** qRT-PCR analysis of the mRNA level of A20 in human primary melanocytes (HPM) and different kinds of melanoma cell lines. **b** Immunoblotting analysis of the protein level of A20 in HPM and different melanoma cell lines. **c** Immunofluorescence staining analysis of the intensity of A20 in mouse melanocytes, B16 melanoma cells and B16F10 melanoma cells. **d** The mRNA level of A20 in nevus and melanoma tissues in Talantov dataset. Data represent the mean ± SEM of triplicates. *P* value was calculated by two-tailed Student’s *t* test. ^*^P < 0.05, ^**^P < 0.01, ^***^P < 0.001, ^****^P < 0.0001. Scale bars = 20 μm. MC melanocyte.

To explore the role of A20 in melanoma, we first examined the mRNA and protein level of A20 in a panel of melanoma cell lines and normal human primary melanocyte (HPM). Through qRT-PCR analysis, we found that the transcriptional levels of A20 were generally increased in melanoma cells compared with melanocytes (Fig. 1a). In addition, the protein levels of A20 were also up-regulated in melanoma cells (Fig. 1b). To further confirm the expression status of A20, we performed immunofluorescence staining on the tails of B6. Cg-Tg (KRT14-Kitl*) 4XTG2Bjl/J mice, which express mutant mouse Kitl cDNA and possess abundant melanocytes in the epidermis, as well as the xenograft tumors of B16 and B16F10 mouse melanoma cells implanted in C57BL/6 mice. The intensity of A20 was markedly increased in B16 and B16F10 cells compared with mouse melanocytes (Fig. 1c). Moreover, we turned to Talantov data set (GSE3189) and found that the mRNA level of A20 was significantly increased in human melanoma specimens compared to benign melanocytic nevus tissues (Fig. 1d). Taken together, the results from in vitro and in vivo demonstrated the up-regulation of A20 in melanoma.

### A20 promotes melanoma growth by regulating cell-cycle progression

**Fig. 2:**
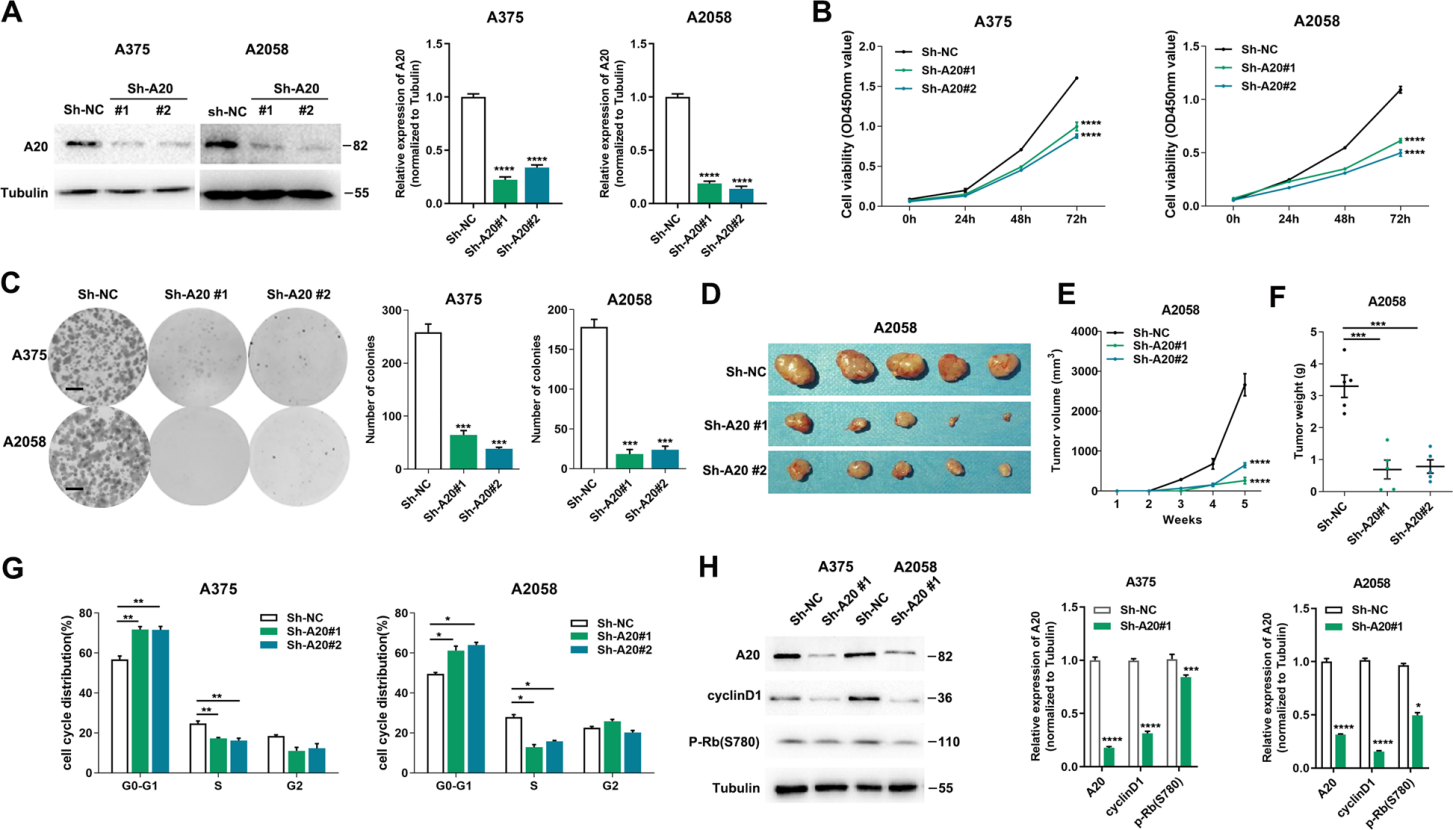
A20 promotes melanoma growth via the regulation of cell-cycle progression. **a** The efficiency of A20 knockdown in melanoma cells examined by immunoblotting analysis. **b** Cell viability of melanoma cells with or without the knockdown of A20 expression. **c** Colony formation assay in two melanoma cell lines with or without the knockdown of A20 expression. **d** The images of excised xenograft tumors with or without the knockdown of A20. **e**, **f** The volumes and the weights of xenograft tumors with or without the knockdown of A20 expression 5 weeks after the subcutaneous implantation. **g** Flow cytometry analysis of cell-cycle distribution of two melanoma cell lines with or without the knockdown of A20 expression. **h** Immunoblotting analysis of the expressions of A20, cyclinD1, phosphor-Rb in two melanoma cell lines with or without the knockdown of A20 expression. Data represent the mean ± SEM of triplicates. *P* value was calculated by two-tailed Student’s *t* test. ^*^P < 0.05, ^**^P < 0.01, ^***^P < 0.001, ^****^P < 0.0001. NC negative control.

We then explored the biological function of A20 in melanoma. Lentiviral transfection was employed to obtain stable knockdown of A20 in both A2058 and A375 cell lines, and the knockdown efficiency was confirmed (Fig. 2a). The knockdown of A20 led to impaired cell proliferation and colony formation in melanoma (Fig. 2b, c). Forwardly, we employed xenograft tumor model to explore whether increased A20 expression contributed to melanoma growth in vivo. A2058 melanoma cells with or without the knockdown of A20 were subcutaneously injected into nude mice. 5 weeks later, the tumor volume and tumor weight were markedly decreased in the A20-knockdown group compared to the control (Fig. 2d–f).

Further, we investigated the mechanism underlying the pro-proliferative role of A20. Previously, several researches have revealed the regulatory role of A20 in various modalities of cell death including cell apoptosis, necroptosis, and pyroptosis^18–20^. Therefore, to figure out whether A20 was involved in the regulation of cell death, we adopted positive controls of melanoma cell apoptosis, necroptosis, and pyroptosis induced by TS (TNF-α and SM-164), TSZ (TNF-α, SM-164 and Z-VAD-FMK), and FC (FeSO4 and CCCP) respectively as described previously^21–23^. TS and TSZ could effectively trigger cell death as revealed by flow cytometry analysis (Fig. S1A), with the expression of apoptosis marker cleaved-Caspase 3 or necroptosis marker phosphor-MLKL increased respectively (Fig. S1B). FC was capable of inducing pyroptosis, manifested as the up-regulation of cleaved-GSDME (GSDME-N-terminal; Fig. S1A, B). However, after the knockdown of A20, we observed neither the increase of cell death, nor the prominent up-regulation of cleaved-Caspase 3, phosphor-MLKL, and cleaved-GSDME (Fig. S1B). Besides, the knockdown of A20 could hardly induce the up-regulation of cleaved-Caspase 3, phosphor-MLKL or cleaved-GSDME in isolated xenograft tumors (Fig. S1C), indicating that cell death might not be involved in the effect of A20 on tumor progression both in vivo and in vitro.

However, the knockdown of A20 could result in significant cell-cycle arrest in both cell lines, with the percentage of cells in G0-G1 phase markedly increased and in S phase decreased (Fig. 2g). In consistent, the expressions of cyclin D and phosphor-Rb were down-regulated after the knockdown of A20 (Fig. 2h). Taken together, A20 altered cell-cycle progression rather than manipulating cell death to promote melanoma cell proliferation in vitro and melanoma growth in vivo.

### A20 contributes to melanoma metastasis by promoting EMT

**Fig. 3:**
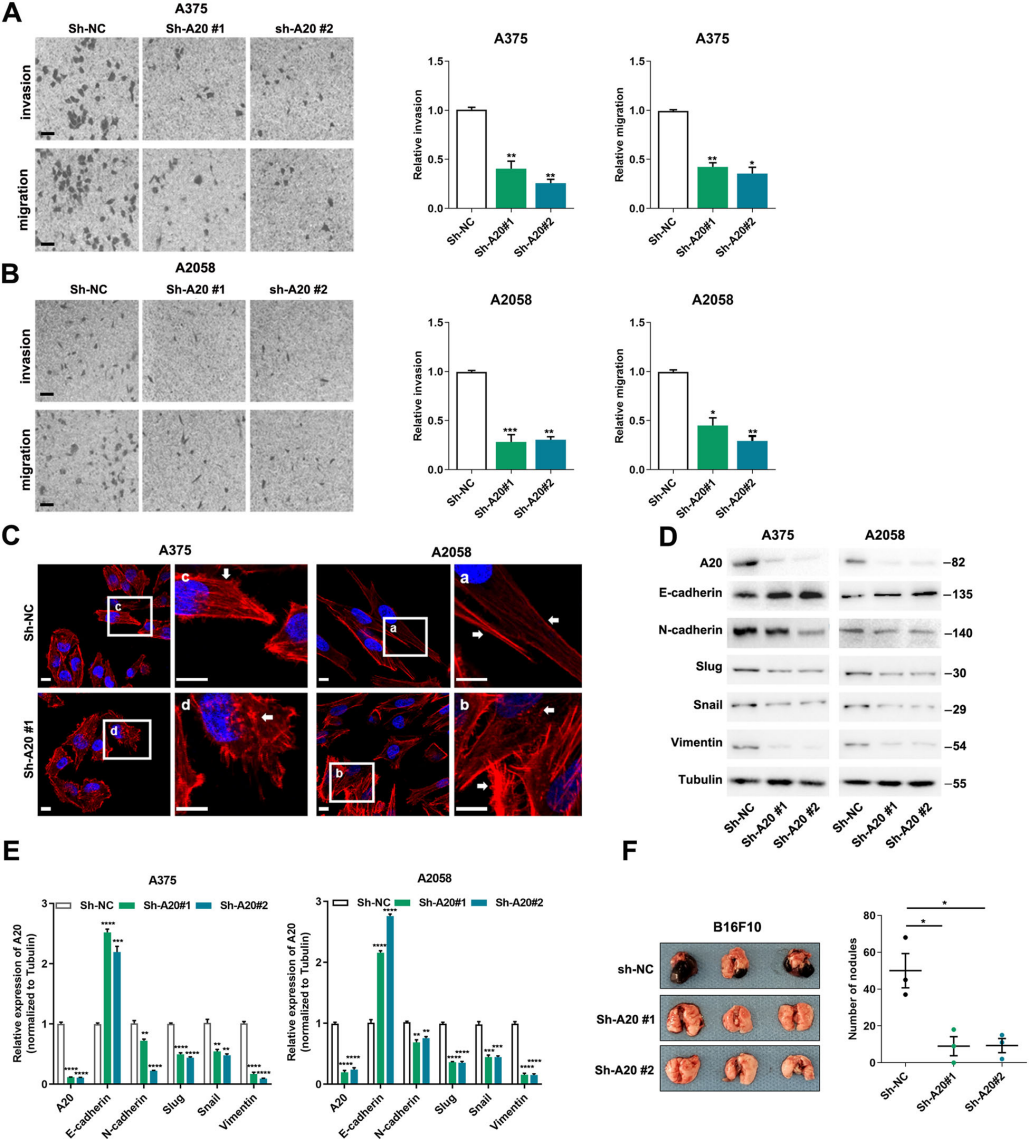
A20 facilitates melanoma metastasis via EMT. **a**, **b** Transwell assay displaying the number of invasive and migrated cells of two cell lines with or without the knockdown of A20. Scale bars = 50 μm. **c** Rhodamine staining showing the arrangement of F-actin in melanoma cells with or without the knockdown of A20. Scale bars = 20 μm. **d**, **e** Immunoblotting analysis of the expressions of EMT-associated molecules with or without the knockdown of A20 expression, and the densitometric analysis of the blots were shown. **f** Number of metastasis nodules on lung in mice model with the caudal vein injection of B16F10 cells with or without the knockdown of A20. Data represent the mean ± SEM of triplicates. *P* value was calculated by two-tailed Student’s *t* test. ^*^P < 0.05, ^**^P < 0.01, ^***^P < 0.001, ^****^P < 0.0001. NC negative control.

Thereafter, we investigated the role of A20 in melanoma metastasis. Through the transwell assay, we found that the knockdown of A20 led to not only impaired invasive ability, but also mitigated migratory capacity (Fig. 3a, b). Previous reports revealed that the control of cellular motility depends critically upon the polymerization and de-polymerization of F-actin, which couples with F-actin arrangement^24^. Therefore, we used rhodamine to stain F-actin to reveal cytoskeleton. Tumor cells in the control group demonstrated smoothly arranged F-actin, while the A20-knockdown group displayed disarrangement and increased breakages in F-actin (Fig. 3c). This might provide the morphological basis for the impaired mobility of A20-knockdown melanoma cell^25^. Of note, the occurrence of tumor metastasis necessitates a series of steps, and epithelial–mesenchymal transition (EMT) is the pivotal one. EMT is highly correlated with the alteration of cellular morphology and is crucial for enhancing cellular motility^26^. After the knockdown of A20, the expressions of EMT process facilitators Slug, Snail, and Vimentin were prominently decreased. Moreover, E-cadherin expression was prominently increased while N-cadherin expression was significantly decreased (Fig. 3d, e), confirming the molecular characteristic of mesenchymal-epithelial transition in case of A20 deficiency. Taken together, A20 contributed to melanoma cell invasion and migration in vitro via the induction of EMT.

We forwardly investigated whether A20 was implicated in tumor metastasis in vivo. B16F10 cells with or without the knockdown of A20 were injected into C57BL/6 mouse caudal vein to establish tumor metastasis model. Three weeks later, all the mice were killed and their lungs were analyzed. As expected, mouse receiving the injection of melanoma cells with the knockdown of A20 exhibited diminished number of lung metastasis (Fig. 3f). Therefore, the up-regulation of A20 contributed to melanoma metastasis in vivo.

### A20 promotes tumor progression through the activation of Akt

**Fig. 4:**
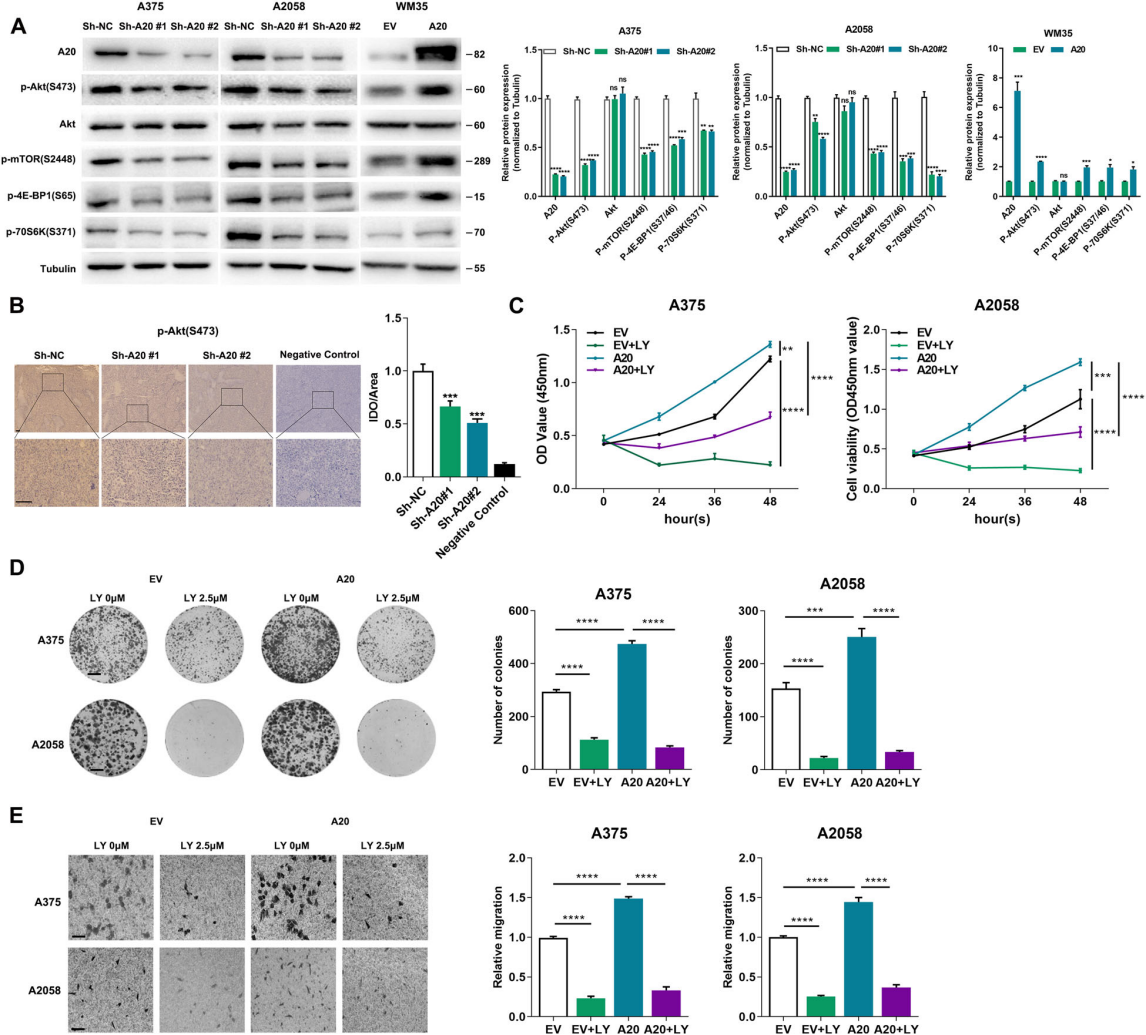
A20 promotes melanoma progression in an Akt-dependent manner. **a** Immunoblotting analysis of the expressions of phosphor-Akt and its downstream proteins in A375 and A2058 cells with or without the knockdown of A20, as well as in WM35 cells with or without the overexpression of A20. *P* value was calculated by two-tailed Student’s *t* test. **b** Immunohistochemical staining analysis of phosphor-Akt in xenograft tumors with or without the knockdown of A20. Scale bars = 100 μm. *P* value was calculated by two-tailed Student’s *t* test. **c** Cell viability of melanoma cells with the intervention of A20 expression and the treatment of 2.5 μM PI3K/Akt inhibitor LY294002. *P* value was calculated by one-way ANOVA. **d** Colony formation of melanoma cells with the intervention of A20 expression and the treatment of 2.5 μM PI3K/Akt inhibitor LY294002. *P* value was calculated by one-way ANOVA. **e** Transwell assay displaying migrated cells with indicated treatment as described in **d**, *P* value was calculated by one-way ANOVA. Data represent the mean ± SEM of triplicates. ^*^P < 0.05, ^**^P < 0.01, ^***^P < 0.001, ^****^P < 0.0001. EV empty vector, NC negative control, LY LY294002.

Next, we wondered the mechanism underlying the oncogenic role of A20. Previous reports have revealed that NF-κB is a canonical downstream of A20 and mediates A20-associated pathomechanism in multiple diseases^27^. Accordingly, we first investigated whether A20 regulated NF-κB to affect melanoma progression. Consistent with previous reports, we observed prominent up-regulation of phosphor-NF-κB p65 after the knockdown of A20 (Fig. S1D), indicating that A20 negatively regulated NF-κB in melanoma. However, NF-κB has been documented to play an oncogenic role in melanoma^28,29^, which does not support the surmise that A20 contributes to melanoma progression via the activation of NF-κB. Therefore, we turned to see whether alternative pathway mediated the oncogenic effect of A20. Of note, Akt is constitutively activated in up to 70% of human melanomas and plays an important role in melanoma pathogenesis^30^. Moreover, Akt activation has exerted combined function on promoting melanoma growth and metastasis via multiple biological activities like cellular metabolism, autophagy, and ROS generation^16,31,32^. After the knockdown of A20, the phosphorylation of Akt at Ser473 was significantly decreased. The phosphorylation of its downstream oncogenic mTOR was also decreased, so were the phosphorylation of both 4E-BP1 and 70S6K that are the substrates of mTOR (Fig. 4a). In contrary, overexpression of A20 in WM35 cells resulted in augmented phosphorylation of these molecules (Fig. 4a). Moreover, we examined the phosphorylation of Akt at Ser473 in previous implanted tumors of A2058 cells through immunohistochemical staining analysis, which revealed that A20 deficiency led to prominent down-regulation of phosphor-Akt in vivo (Fig. 4b).

We then wondered whether the tumorigenic effect of A20 was mediated by Akt. Melanoma cells were transfected with A20 overexpression plasmid and then treated with PI3K/Akt inhibitor LY294002. The mono-treatment with LY294002 could suppress the proliferation of melanoma cells, which was consistent with previous reports (Fig. 4c)^33^. Moreover, LY294002 could reverse the promotive influence of A20 on melanoma cell proliferation and colony formation (Fig. 4c, d). We also performed transwell assay and found that LY294002 treatment could reverse the potentiated migration of melanoma cells caused by A20 (Fig. 4e). Taken together, the oncogenic effect of A20 on melanoma cell proliferation and migration was dependent on Akt pathway.

### A20 potentiates glycolysis via Akt in melanoma

**Fig. 5:**
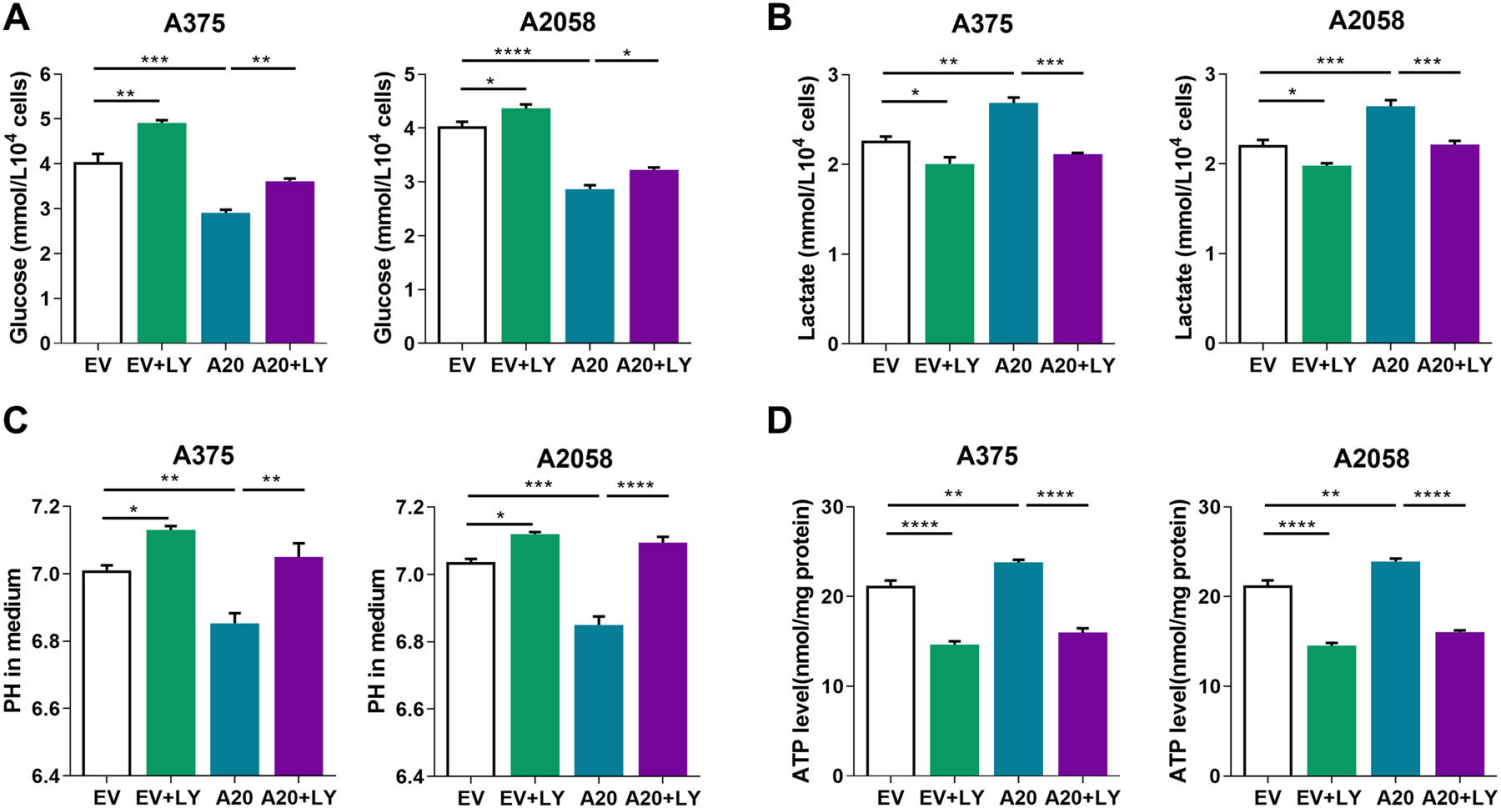
A20 potentiates glycolysis in melanoma cell. **a**, **b** Glucose and lactate concentration (in the culture media) of melanoma cells with the intervention of A20 expression and with/without the treatment of 2.5 μM PI3K/Akt inhibitor LY294002 after 48 h of culture. **c** Culture medium PH value of melanoma cells with the intervention of A20 expression and with/without the treatment of 2.5 μM PI3K/Akt inhibitor LY294002 after 48 h of culture. **d** Intracellular ATP levels in melanoma cells with indicated interventions as described in **c**. Data represent the mean ± SEM of triplicates. *P* value was calculated by one-way ANOVA. ^*^P < 0.05, ^**^P < 0.01, ^***^P < 0.001, ^****^P < 0.0001. EV empty vector, LY LY294002.

The oncogenic effect of Akt can be attributed to multiple biological alterations, among which glycolysis provides sufficient energy and intermediate metabolite for the rapid proliferation of tumor cells^32^. Given this, we wondered whether A20 could induce metabolic rewiring in melanoma, and, if possible, whether this effect was associated with Akt activation. The overexpression of A20 led to decreased glucose and increased lactate in the culture medium, which was suppressed by the additional treatment of LY294002 (Fig. 5a, b), confirming that the glycolytic effect of A20 was dependent on Akt. In line with this, the PH value of the culture medium was prominently decreased after A20 overexpression, whereas increased after the co-treatment with LY294002 (Fig. 5c). Since that the activation of glycolysis supplies the energy for the rapid proliferation of tumor cell^17^, we testified the intracellular ATP level after the intervention of A20 expression. The overexpression of A20 significantly increased the production of ATP, whereas the inhibition of Akt reversed this effect (Fig. 5d). Taken together, A20 could potentiate glycolysis via Akt pathway in melanoma, which was associated with increased ATP production and rapid cell proliferation.

**Fig. 6:**
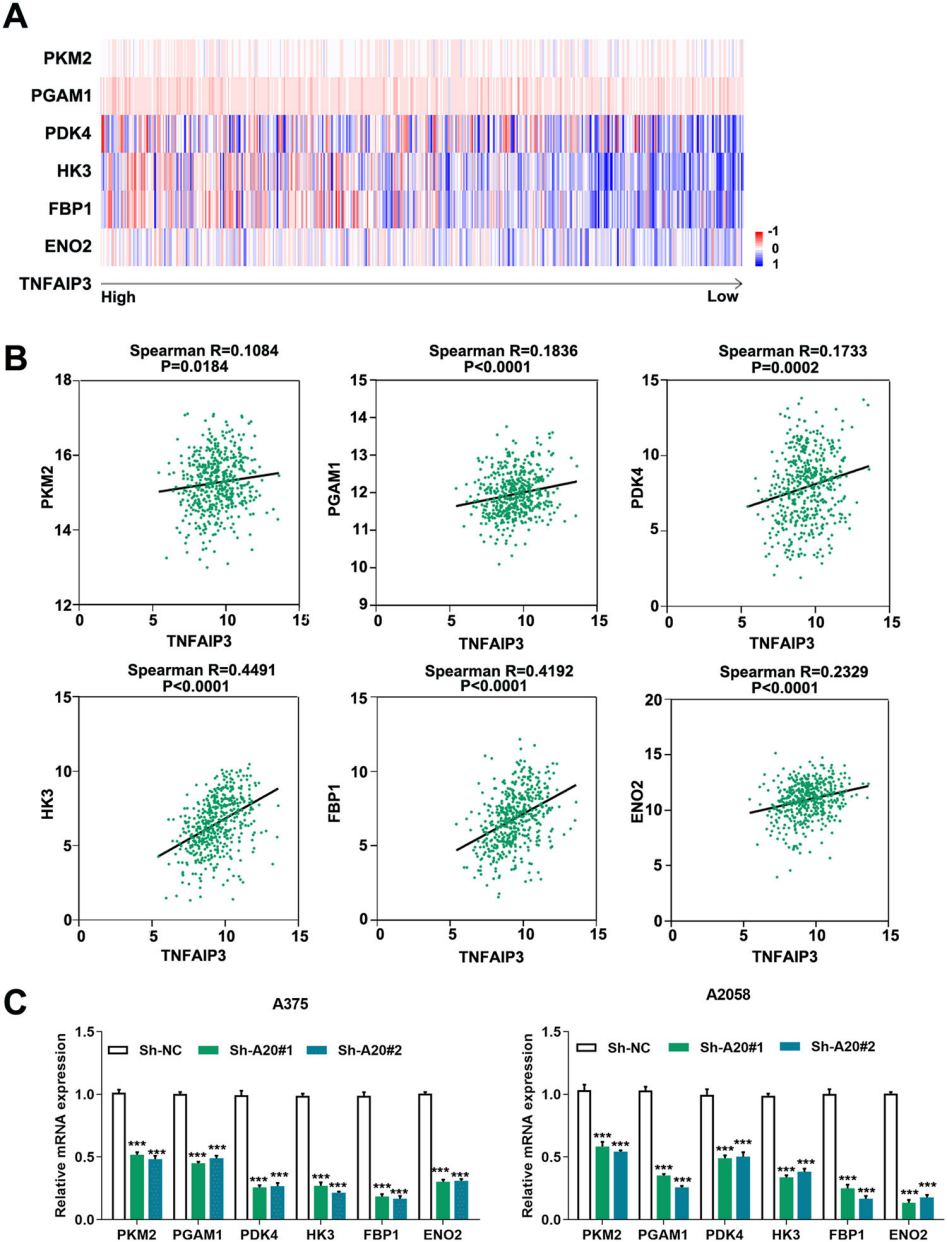
A20 regulates the expressions of multiple glycolytic genes in melanoma. **a** The heatmap of the expressions of *TNFAIP3* (encoding A20) and multiple glycolytic molecules in TCGA SKCM database. **b** The correlation analysis between and several glycolytic molecules in TCGA SKCM database. **c** qRT-PCR analysis of the transcriptional level of the glycolytic molecules of HK3, PKM2, FBP1, PGAM1, ENO2, and PDK4 after the knockdown of A20 in both of the two cell lines. Data represent the mean ± SEM of triplicates. *P* value was calculated by two-tailed Student’s *t* test. ^***^P < 0.001. NC negative control.

We then wondered which genes downstream of Akt were responsible for the potentiation of glycolysis by A20. Through TCGA SKCM database, we systematically analyzed the relationship between A20 and multiple glycolytic molecules. The mRNA level of A20 was in positive correlation with the mRNA expressions of PKM2, PGAM1, PDK4, HK3, FBP1, and ENO2 respectively (Fig. 6a, b). Moreover, after the knockdown of A20, the transcriptional levels of PKM2, PGAM1, PDK4, HK3, FBP1, and ENO2 were generally down-regulated (Fig. 6c). Therefore, A20 might promote glycolysis via the regulation of multiple genes in melanoma.

### A20 contributed to the acquired resistance to Vemurafenib via the activation of Akt

**Fig. 7:**
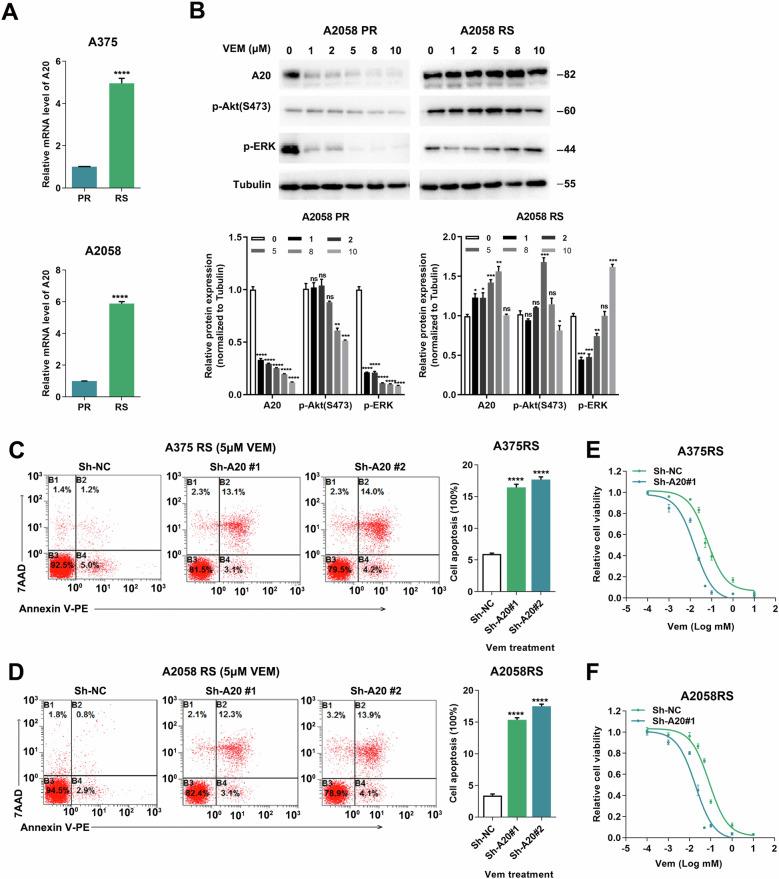
A20 confers the acquired resistance to Vemurafenib treatment by regulating Akt. **a** The relative mRNA level of A20 in parental and A375RS and A2058RS melanoma cells in response to 5 μM Vemurafenib treatment. **b** The protein expressions of A20, phosphor-Akt and phosphor-ERK in parental and A2058RS melanoma cells in response to Vemurafenib treatment. **c**, **d** Flow cytometry analysis of cell apoptosis of A375RS and A2058RS melanoma cells with or without the knockdown of A20 in response to 5 μM Vemurafenib treatment after 24 h. **e**, **f** The relative cell viability of A375RS and A2058RS melanoma cells with or without the knockdown of A20 after the treatment with indicated concentrations of Vemurafenib after 24 h. Data represent the mean ± SEM of triplicates. *P* value was calculated by two-tailed Student’s *t* test. ^*^P < 0.05, ^**^P < 0.01, ^***^P < 0.001, ^****^P < 0.0001. NC negative control, PR parental, RS resistance, VEM Vemurafenib.

Accumulative evidence has revealed that the activation of Akt was greatly involved in the acquired resistance to Vemurafenib^34^. Hence, we speculated that A20 might regulate the acquired resistance to Vemurafenib via Akt. Through the chronic treatment with Vemurafenib as previously described^35^, our group has already established the model of acquired resistance in both *BRAF*-mutant A375 cell line (A375RS) and A2058 cell line (A2058RS) before, which was characterized by the measurement of cell viability under gradually increased concentrations of Vemurafenib treatment (data not shown)^36^. The transcriptional level of A20 was significantly elevated in A375RS and A2058RS melanoma cells as compared with parental cells (Fig. 7a). Moreover, Vemurafenib treatment caused dose-dependent decreases in the expression of p-ERK in parental cells, whereas the resistant cells (A2058RS) maintained elevated levels of p-ERK (Fig. 7b). In line with previous reports^37^, Akt phosphorylation was increased more prominently in A2058RS cells compared with parental cells (Fig. 7b). While Vemurafenib treatment resulted in dose-dependent decrease of A20 expression in parental cells, the resistant cells (A2058RS) maintained high expression of A20 as the concentration of Vemurafenib gradually increased (Fig. 7b). Then, we obtained the knockdown of A20 to see the effect on the resistance to targeted therapy. While Vemurafenib had little impact on the apoptosis of A375RS and A2058RS cells, A20 expression deficiency induced more apoptotic cells (Fig. 7c, d). Moreover, A20 suppression significantly increased the sensitivity of A375RS and A2058RS cells to Vemurafenib treatment revealed by the CCK8 assay (Fig. 7e, f). After the knockdown of A20, the activation of Akt was attenuated prominently in A2058RS cells (Fig. S1E). To forwardly support that the activation of Akt and glycolysis was implicated in the acquired resistance, we examined whether the inhibition of Akt or glycolysis could re-sensitize resistant melanoma cells to Vemurafenib treatment. As was shown, the combined treatment with Akt inhibitor LY294002 or glycolysis inhibitor 2-DG profoundly attenuated the cell viability of A375RS and A2058RS cells in response to Vemurafenib treatment (Fig. S1F). In summary, the up-regulation of A20 contributed to the acquired resistance to Vemurafenib via the activation of Akt, and targeting A20 could be employed as a potential approach to increase the therapeutic efficacy of *BRAF*-targeted therapy.

## Discussion

In the present study, we first found that A20 expression was up-regulated in melanoma. Subsequent functional study proved that A20 contributed to melanoma cell proliferation in vitro and tumor growth in vivo by regulating cell-cycle progression. Moreover, A20 facilitated melanoma cell invasion and migration in vitro and tumor metastasis in vivo by mediating EMT. Mechanistically, Akt-dependent potentiation of glycolysis and the up-regulation of several glycolytic enzymes were responsible for the role of A20 in melanoma progression. Furthermore, we uncovered that A20-mediated Akt activation was also implicated in rendering acquired resistance to Vemurafenib in *BRAF*-mutant melanoma. To sum up, up-regulated A20 played an oncogenic role in melanoma by promoting the activation of Akt (Fig. S2).

A20 is regarded as a primary responsive gene induced by TNF-α and a crucial negative mediator of inflammation and immune response via the regulation of downstream NF-κB^38^. Therefore, previous studies mainly focused on the role of A20 in autoimmune diseases^39^. Recently, accumulative evidence has revealed that A20 was also crucial for carcinogenesis and cancer progression. A20 was identified as a tumor suppressor gene in Hodgkin lymphoma and primary mediastinal B-cell lymphoma. The genetic inactivation or mutation of *TNFAIP3* led to constitute activation of NF-κB to enable tumor cell survival^40,41^. Furthermore, the genetic deletion, promoter methylation and genetic mutation of *TNFAIP3* also frequently occurred in mantle cell lymphoma and diffuse large B-cell lymphoma to facilitate the tumor development^42^, indicating that A20 exerted tumor suppressive effect. In contrary, A20 expression was up-regulated in human basal-like breast cancer and facilitated epithelial–mesenchymal transition to promote cancer metastasis^7^. Besides, targeting A20 was capable of suppressing glioma stem cell survival and restraining tumor growth^43^. These reports emphasized that the pathogenic role of A20 was cancer type-specific and tumor context-dependent. Herein, we for the first time discovered the up-regulation of A20 in melanoma, and proved that A20 was a novel oncogenic factor for melanoma via the simultaneous contribution to tumor growth and metastasis, demonstrating the great potential of A20 as a valuable therapeutic target.

Dysregulated A20 expression was tightly associated with various aspects of tumor biology. In T-cell acute lymphoblastic leukemia, the restoration of A20 in miR-125b-overexpressing cells could efficiently decreased glucose uptake and oxygen consumption to induce the differentiation of leukemia cell^44^. A20 could also control the self-renewal of cancer stem cell by defending against TNF-α-induced cell apoptosis in glioma^40^. Moreover, the genetic deletion of A20 in hepatocyte resulted in chronic liver inflammation and increased the susceptibility to chemical or high fat-diet-induced hepatocellular carcinoma development^45^. Therefore, A20 was a versatile molecular with integrated function of regulating metabolic rewiring, stem cell self-renewal and cancer-related inflammation in tumor. Apart from this, we proved that A20 played a crucial role in glycolysis, with the expressions of a series of glycolytic enzymes significantly decreased after the knockdown of A20. This finding was in contrary to two previous reports demonstrating that A20 exerted negative impact on glycolysis in hepatocellular carcinoma and leukemia^44,46^. The discrepancy may be due to the heterogeneity of distinct tumor types, which indicated that tumor context should be taken into consideration for A20-based metabolism intervention in cancer therapy.

Akt is constitutively activated in up to 70% of human melanomas and plays an important role in melanoma pathogenesis^47^. This is frequently associated with the down-regulation and loss of PTEN mediated by genetic or epigenetic mechanisms^48^. In some subsets of melanomas, the activating mutations in *NRAS*, *PIK3CA* and *c-KIT* were also related with the hyper-activation of Akt^49,50^. Moreover, the down-regulation of PIB5PA was reported to promote Akt activation independent of PTEN by regulating intracellular 5-phosphatase content in melanoma^51^. Extending to these mechanisms underlying Akt activation, we identified A20 as a novel mediator of Akt. It has been reported that A20 potentiated TGF-β signaling to promote the metastasis of basal-like breast cancers^7^. Besides, TGF-β was capable of activating PI3K-Akt signaling to drive prostate cancer cell migration^52^. Therefore, it is of possibility that A20 may activate Akt in melanoma via the activation of TGF-β, which would be clarified further in our future study. Notably, a previous study has revealed that the intervention of A20 showed little impact on Akt activation in T cell^53^, which was different from the results in the present study. The discrepancy could be associated with the heterogeneity of different kind of cells, and the regulatory role of A20 in Akt pathway may be specific to malignancy rather than normal cell.

The suppression of MAPK pathway by targeting *BRAF* has achieved revolutionary therapeutic progress in *BRAF*-mutant melanoma. However, the acquired resistance would inevitably occur via heterogeneous mechanisms, which prominently hindered the efficacy of targeted therapy and worsened the prognosis of patients^54^. To date, extensive mechanisms have been documented to render the acquired resistance, including the aberrations in PI3K/Akt pathway^54^. Specifically, the activation of PI3K/Akt signaling could be induced by several approaches like the up-regulation of IGF1R and the activating mutations in *PI3K* and *Akt*^55–57^. In addition to these, the up-regulation of A20 was also responsible for the aberrant activation of Akt in melanomas with acquired resistance, providing alternative targets for increasing the efficacy of targeted therapy. Of note, the up-regulation of A20 has been reported to be greatly involved in conferring the resistance to chemotherapy, DNA-damaging therapy, and TRAIL-based therapy^58–60^. Therefore, A20 is a versatile regulator and a promising target for the determination of the treatment outcomes of diverse therapeutic approaches in cancer.

In summary, our study demonstrates that A20 is remarkably up-regulated in melanoma and plays an oncogenic role by simultaneously promoting tumor growth and metastasis, and rendering the acquired resistance to *BRAF*-targeted therapy. Therefore, A20 is a valuable target for restraining melanoma development and optimizing the efficacy of targeted therapy, which needs further investigation in future clinical trials.

## Materials and methods

### Statistical analysis

All experiments were repeated at least three times unless otherwise indicated. Error bars represent standard error of the mean (SEM). Student’s *t* test was used to compare two groups of independent samples. The differences among multiple groups were analyzed using one-way ANOVA. Liner regression was used to confirm the correlation between two groups of dependent samples. *P* value < 0.05 was considered statistically significant. All statistical analyses were performed with GraphPad Prism (GraphPad software 6.0).

## Supplementary information

Supplementary Information

Supplementary Figure S1

Supplementary Figure S2
