# Correction: A20 promotes melanoma progression via the activation of Akt pathway

**DOI:** 10.1038/s41419-025-08121-x

**Published:** 2025-11-20

**Authors:** Jinyuan Ma, Huina Wang, Sen Guo, Xiuli Yi, Tao Zhao, Yu Liu, Qiong Shi, Tianwen Gao, Chunying Li, Weinan Guo

**Affiliations:** Department of Dermatology, Xijing Hospital, Fourth Military Medical University, No 127 of West Changle Road, 710032 Xi’an, Shaanxi China

Correction to: *Cell Death and Disease* 10.1038/s41419-020-03001-y, published online 23 September 2020

The authors apologize for the errors occurred during the assembly of Figure 2C, in which the representative image of colony formation of A2058 sh-NC group was placed incorrectly. We have revised the errors and shown the corrected images in the corrected Figure 2C. All of our authors confirmed that this revision will not change our results and conclusions. The authors would like to apologize for any inconvenience caused.

**Figure 2C original** 
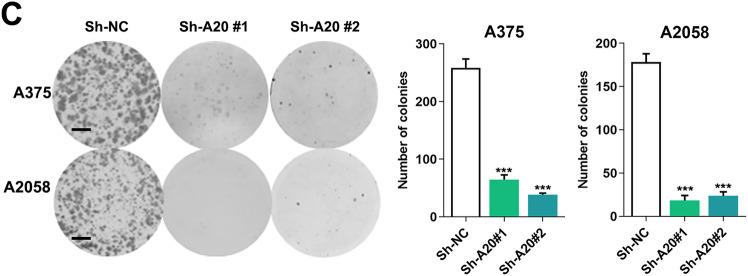


**Figure 2C amended** 
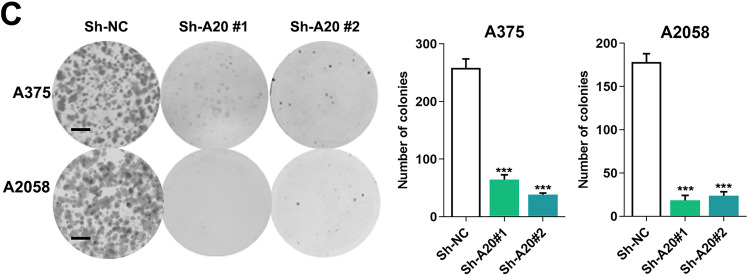


The original article has been corrected.

