# Supplementary material for: A20 promotes melanoma progression via the activation of Akt pathway
Source: Cell Death Dis. 2020 Sep 23;11(9):794. doi: 10.1038/s41419-020-03001-y (PMC7511359; doi:10.1038/s41419-020-03001-y)
Supplement: Supplementary file 1 — Supplementary Information [file 41419_2020_3001_MOESM1_ESM.docx]

**Supplementary Materials and Methods**

**Human cells and reagents**

Human Primary Melanocytes (HPMs) that were isolated from human foreskin specimens obtained during circumcision surgery were cultured in Medium 254 (Invitrogen) supplemented with Human Melanocyte Growth Supplement (Gibco). Human melanoma cell lines A375, A2058, WM35 and mouse melanoma cell line B16F10 were grown in DMEM (Gibco 11995073). Human melanoma cell lines WM793B, 451LU, UACC62, HTB140, UACC257 and mouse melanoma cell line B16 (Chinese Academy of Sciences, Shanghai, TCM2) were grown in RPMI (Gibco C11875500BT). All the cultured cells were supplemented with 10% fetal bovine serum (Gibco 10099-141) and 1% Pen Strep Glutamine (Gibco 10378-016). Cells were grown at 37°C in 5% CO_2_ and lifted with 0.05% Trypsin-EDTA (Gibco 25300-054). All these melanoma cell lines were authenticated by short-tandem repeat (STR) fingerprinting in center of DNA typing in Fourth Military Medical University in 2016 and tested for mycoplasma contamination.

To generate cell line with acquired resistance, *BRAF*V600E-mutant cell lines A2058 and A375 were treated with different concentrations (approximately 3×IC_50_, 5×IC_50_, 10×IC_50_ of the parental sensitive cell line) of Vemurafenib for 2 to 3 months until a subline grew progressively as described^49^. The resistant cell lines were designated A375RS and A2058RS.

Vemurafenib, LY294002, CCCP and 2-DG were purchased from Selleck. FeSO_4_ was purchased from Sigma. The apoptosis and necroptosis of melanoma cell was induced by TS (TNF-α and SM-164) and TSZ (TNF-α, SM-164 and Z-VAD-FMK) respectively according to the manufacturer’s protocol of the Apoptosis induction Kit (C0006S, Beyotime Biotechnology, China) and Necroptosis induction Kit (C1058S, Beyotime Biotechnology, China).

The research protocol was designed and executed according to the principles of the Declaration of Helsinki and was approved by the ethics review board of Fourth Military Medical University.

**Real-Time PCR**

Total RNA was extracted by RNAiso Plus (9109; Takara; Kyoto; Japan). Isolated RNA was reversely transcripted into cDNA using the First Strand cDNA Synthesis Kit (Takara) according to the manufacturer’s instruction. Subsequent gene expression was then analyzed using SYBR Select Master Mix (Takara 639676). The primers used were listed in Table S1. The relative gene expression levels were normalized to human β-actin levels and calculated using the comparative Ct (2-^ΔΔCT^) method.

**Plasmid vectors and siRNA transfection**

Small interfering RNA (GenePhama, Shanghai, China) was used to knockdown A20 expression. Lipofectamine 3000 transfection reagent kit (Invitrogen L3000-015) was used for transfection according to the manufacturer’s recommended procedures. Short-harpin RNA (shRNA, GenePhama, Shanghai, China) was used to obtain the knockdown of A20 in A375, A2058 and B16F10 melanoma cells with the use of Lipofectamine 3000 transfection reagent kit. After 72 hrs of transfection, puromycin (abcam, ab141453) was used to remove the cells with no transfection efficiency. The A20 overexpression plasmid (A20 plasmid), as well as empty plasmid vectors were designed and made by GenePharma (Shanghai, China), and transferred into melanoma cells with Lipofectamine 3000 transfection reagent kit according to the manufacturer’s recommended procedures. Puromycin (abcam) was used to remove the non-transfected cells. The sequences of shRNAs against human *A20* are used as the same as previous studies^1^. Moreover, the sequences of shRNA against mouse A20 are used as the same as a previous study^2^.

**Immunohistochemical staining analysis**

Melanoma xenograft tumors isolated from mice were fixed in 10% neutral buffered formalin and paraffin-embedded. 2μm FFPE consecutive tumor sections were stained with Hematoxylin (Dako) and Eosin G (Dako). For immunohistochemical staining analysis, heat-mediated antigen retrieval was performed in citrate buffer at pH 6.0 or EDTA at pH 8.0. The dilution of indicated antibody was as follows: anti-human p-Akt (S473) monoclonal rabbit antibody (1:200; ab81283; abcam), anti-human cleaved-caspase 3 polyclonal rabbit antibody (1:200; #9661, Cell Signaling Technology), anti-human GSDME-N-terminal monoclonal rabbit antibody (1:200; ab215191; abcam), anti-human phosphor-MLKL monoclonal rabbit antibody (1:250; ab187091; abcam). Images were quantified using Image J Proplus software.

**Mouse xenograft study**

Cg-Tg (KRT14-Kitl*)4XTG2Bjl/J mice were bought from the Jackson Laboratory. All mice were maintained under pathogen-free conditions at the Fourth Military Medical University, Xi’an, China, and were at the age of 4-6 weeks at the time of cell implantation. Mice were kept at 12 hrs/12 hrs light cycle and received standard food and water. All animal studies and experimental procedures were approved by the Animal Care and Use Committee of the animal facility at the Fourth Military Medical University. The experimental design and number of mice assigned to each treatment were based on prior experience with similar models and provided sufficient statistical power to discern significant differences.

For the observation of the effect of A20 on melanoma growth *in vivo*, five-week-old female NOD/SCID nude mice were randomly divided into the three groups, then receiving subcutaneous injection of 5 × 10^6^ A2058 melanoma cells with or without the knockdown of A20 into the back of nude mice (n = 5 for each group). Mice were examined of the volumes of the tumors at indicated time points. Five weeks later, the mice were sacrificed and the tumors were harvested and photographed, and the weights of tumors were examined as well. The comparisons of tumor volumes and tumor weights between different groups were analyzed by two-tailed Student’s unpaired *t* test.

For the observation of the effect of A20 on melanoma metastasis *in vivo*, C57BL/6 mice were randomly divided into the three groups, receiving the [caudal vein](D:/Users/lenovo/AppData/Local/Youdao/Dict/7.5.0.0/resultui/dict/javascript:;) injection of 3×10^6^ B16F10 cells with or without the knockdown of A20 (n = 3 for each group), and lungs derived from model mice were observed after 3 weeks and recorded with number of the metastatic lesions.

**Western blot**

Western blot was performed as described previously^3^. In brief, samples were separated with SDS-PAGE, transferred to polyvinylidene difluoride membrane and probed with the corresponding antibodies. The dilution of antibodies were as follows: anti-human A20 monoclonal rabbit antibody (1:1000; ab92324; abcam), anti-human/mouse p-Akt (S473) monoclonal rabbit antibody (1:1000; ab81283; abcam), anti-human/mouse pan Akt polyclonal rabbit antibody (1:1000; ab18785; abcam), anti-human/mouse snail monoclonal rabbit antibody (1:1000; #3879; CST), anti-human/mouse slug monoclonal rabbit antibody (1:1000; #9585; CST), anti-human/mouse Vimentin monoclonal rabbit antibody (1:1000; #5741; CST), anti-human/mouse cyclin D1 monoclonal rabbit antibody (1:1000; #55506; CST), anti-human/mouse p-Rb (S780) polyclonal rabbit antibody (1:1000; ab47763; abcam), anti-human/mouse p-mTOR (S2448) monoconal rabbit antibody (1:1000; ab109268; abcam), anti-human/mouse p-4E-BP1 (S65) rabbit antibody (1:1000; #2855; CST), anti-human/mouse p-70S6K (Ser371) rabbit antibody (1:1000; #9208; CST), anti-human/mouse E-cadherin mouse antibody (1:1000; #14472; CST), anti-human/mouse N-cadherin rabbit antibody (1:1000; #13116; CST), anti-p-ERK rabbit monoclonal (1:2000, ab201015, abcam), anti-human cleaved-caspase 3 polyclonal rabbit antibody (1:1000; #9661, Cell Signaling Technology), anti-human GSDME-N-terminal monoclonal rabbit antibody (1:1000; ab215191; abcam), anti-human phosphor-MLKL monoclonal rabbit antibody (1:1000; ab187091; abcam), anti-human NF-κB p65 monoclonal rabbit antibody (1:1000; #8242; CST), anti-human phosphor-NF-κB p65 monoclonal rabbit antibody (1:1000; #3033; CST), anti-human/mouse Tubulin polyclonal rabbit antibody (1:1000; ab6046; abcam), anti-mouse and anti-rabbit secondary antibodies (1:3000; 115-035-003; Jackson ImmunoResearch, West Grove, PA ). Signals were detected using Western ECL Substrate (Thermo Scientific).

**Immunofluorescence staining analysis**

Cells were grown on sterile glass coverslips, rinsed with PBS, fixed in 4% paraformaldehyde for 20 min and permeabilized using 0.5% Triton X-100 in PBS for 5 mins. Cells were blocked in 3% BSA+0.1% Triton in PBS for 1 hr, incubated with primary antibodies (anti-human/mouse A20 1:200, ab13597, abcam; anti-Melan-A 1:200, ab51061, abcam); for 1 hr at room temperature, washed and probed with the secondary antibodies conjugated to Alexa Fluor 488 (Invitrogen A11001) and Alexa Fluor 568 (Invitrogen A11001), Staining of F-actin was performed with BODIPY FL phallacidin (Invitrogen), for 1 hr and were stained with DAPI (ROCHE 10236276001). Images were obtained using an inverted confocal laser scanning microscope (Zeiss).

**Flow cytometry analysis of cell death**

A375/A2058 cell lines pre-seeded (2.5×10^5^ cells) in 6-wells plate (Costar) were transfected with siRNA for 72 hrs and were then collected and stained by using Annexin V-PI double staining assay kit (C1062M, Beyotime Biotechnology, China) according to manufacturer’s instructions. Stained samples were acquired using a Beckman Coulters Gallios flow cytometer. Data were analyzed by FlowJo V10 software. Experiments were repeated at least in triplicate. For A375RS/A2058RS cells transfected with shRNA, Annexin V-PE/7AAD double staining assay kit (CA1030, Solarbio Life Sciences, China) was used according to manufacturer’s instructions.

**Cell proliferation analysis**

Melanoma cells were plated in 96-well plates at a density of 5000 cells/well incubated at 37°C under a 5% CO2 atmosphere. Cell viability was assessed after 0h, 24h, 48h and 72h respectively using a Cell Counting kit-8 (MCE) according to the manufacturer’s instructions. Ultraviolet spectrophotometer was used to detect percentage of live cell and cell viability was calculated with the absorbency.

**Colony formation assay**

A375 and A2058 cells tranfected with negative control shRNA or A20 shRNA were seeded to six-well plates at a density of 2000 cells/well and incubate for 14 days. Then the colonies were fixed and stained by [crystal violet](D:/Users/lenovo/AppData/Local/Youdao/Dict/7.5.0.0/resultui/dict/javascript:;) (Sigma) for 30 min. The colonies were observed and photographed after washing and drying up the plates.

**Transwell assay**

Transwell assay were applied using 24-well insert, 8 μm pore size. In brief, 500 μL DMEM medium supplemented with 10% FBS was loaded into the lower side of the Transwell chamber, while 2 × 10^4^ cells in 200 μl FBS-free DMEM medium were loaded into the upper side. 24 h later, cells penetrated to the underside of the membrane were fixed and stained, and further counted in 5 random fields under a microscope. For invasion assays, transwell chambers with 8 μm-pore size membrane filter inserts (Corning) coated with Matrigel (BD Biosciences) were used to determine cell invasion. The non-motile or non-invasive cells on the upper side of the filter were removed, while the motile or invasive cells on the lower side were stained with crystal violet. For the quantification of the invasive and migratory cells, we used the “Multi-point” tool in ImageJ software to count the stained cells. Five fields for each well were counted under the inverted system microscope (Ti-S, Nikon).

**Glucose consumption, lactate production, PH value and ATP level**

The glucose and lactate in the culture media was examined by using Glucose Colorimetric Assay Kit II and Lactate Colorimetric Assay Kit II (BioVision Research Products) respectively according to the manufacturer’s instructions. Briefly, equal number of cells with indicated treatment were seeded in 6-well plates and cultured in phenol-red free DMEM for 48 hours. Cultured medium was then mixed with the reaction solution. Glucose and lactate levels were measured at 450 nm using a Model 680 Microplate Reader. Cells were lysed and protein concentration was measured using BCA protein assay kit and values were normalized to cellular protein concentration.

Intracellular ATP levels were determined using the ATP Bioluminescence Assay Kit (S0026, Beyotime, Shanghai, China) according to the manufacturer’s instructions. Briefly, equal number of cells was seeded in 6-well plates and cell lysates were diluted appropriately in reaction buffer. The levels of ATP were determined by mixing 50 μl of the supernatant with 50 μl of luciferase reagent, which catalyzed the light production from ATP and luciferin. The emitted light was linearly related with the ATP concentration and measured using a microplate reader (Model 680, Bio-Rad). Protein concentration was measured using BCA protein assay kit and all values were normalized to cellular protein concentration.

**Cell Cycle analysis**

For cell cycle analysis, 2.5×10^5^ cells transfected with negative control shRNA or shRNA against A20 were synchronized by serum starvation for 48 hours as previously described ^4^, followed by the culture in 6-well-plate for another 48 hours prior to cell cycle analysis. Melanoma cells were harvested by trypsinization (Solarbio) and then fixed in 70% (v/v) cold ethanol at 4°C overnight. After washing with ice-cold PBS, the fixed-cell pellets were collected by centrifugation and re-suspended in PI/RNase Staining Buffer for staining of DNA and finally analyzed by Becton Dickinson FACScan. More importantly, to rule out the effect of puromycin on cell-cycle progression^5^, melanoma cells transfected with negative control shRNA or shRNA against A20 were recovered from puromycin treatment for 72 hours before proceeding to cell cycle analysis.

**Supplementary Figure Legends**

**Supplementary Figure S1. (A)** Flow cytometry analysis of cell death in two melanoma cell lines with or without the knockdown of A20 expression at 72hrs. Melanoma cell apoptosis, necroptosis and pyroptosis were induced by TS (TNF-α and SM-164), TSZ (TNF-α, SM-164 and Z-VAD-FMK) and FC (FeSO4 (100μM) combined with CCCP (20μM)) respectively. Data represent the mean ± SEM of triplicates. *P* value was calculated by two-tailed Student’s *t*-test. **(B)** Immunoblotting analysis of cleaved-Caspase 3, phosphor-MLKL and cleaved-GSDME were examined in two melanoma cell lines with or without the knockdown of A20 expression, and in the positive controls of cell apoptosis, necroptosis and pyroptosis induced by TS (TNF-α and SM-164), TSZ (TNF-α, SM-164 and Z-VAD-FMK) and FC (FeSO4 (100μM) combined with CCCP (20μM)) respectively. **(C)** Immunohistochemical staining analysis of cleaved-Caspase 3, phosphor-MLKL and cleaved-GSDME in isolated xenograft tumors with or without the knockdown of A20. **(D)** Immunoblotting analysis of phosphor-NF-κB p65 and NF-κB p65 with or without the knockdown of A20 expression in A375 melanoma cells. **(E)** Immunoblotting analysis of phosphor-Akt (S473) with or without the knockdown of A20 expression in A2058RS cells. **(F)** Relative cell viability of A375RS and A2058RS melanoma cells after the combined treatment with 2.5μM PI3K/Akt inhibitor LY294002 or 5mM 2-DG in response to 5μm Vemurafenib.

**Supplementary Figure S2.** Schematic presentation of the oncogenic role of A20 in melanoma. Up-regulated A20 potentiates Akt signaling and glycolysis to promote melanoma cell proliferation and metastasis. In addition, A20 also contributes to the acquired resistance to Vemurafenib via the regulation of Akt.

**Supplementary Table**

**Supplementary Table S1.** Primers used for real time PCR assays.

| **Gene** | **Direction** | **Primer sequence** |
| --- | --- | --- |
| *TNFAIP3* | Forward | CCGGTCTCTGTTAACA AGTGG |
|  | Reverse | GAGTGTTACAG ATATCCCATCGTC |
| *PKM2* | Forward | ATGTCGAAGCCCCATAGTGAA |
|  | Reverse | TGGGTGGTGAATCAATGTCCA |
| *PDK4* | Forward | TCCCCGCTGTCCATGAAG |
|  | Reverse | CGTTCTTTCACAGGCATTTTCTG |
| *FBP1* | Forward | ACATCGATTGCCTTGTGTCC |
|  | Reverse | CCACCAAAATGAACTCCCCG |
| *HK3* | Forward | GGGCTACTTGGGGATTGCTTCACA |
|  | Reverse | ACGTGTCTTTCTCAAGGCCCAGC |
| *PGAM1* | Forward | ATGCTAAGCCATGACCAGTGAG |
|  | Reverse | ATCACCACGCAGGTTACATTCG |
| *ENO2* | Forward | AATGGATGTTGCCGCTTCAGAGTTC |
|  | Reverse | TAAGTCAGCAATGAATGTGTCCTCG |
|  |  |  |

**Reference**

1. J. A. Vendrell, S. Ghayad, S. Ben-Larbi, C. Dumontet, N. Mechti, P. A. Cohen, A20/TNFAIP3, a new estrogen-regulated gene that confers tamoxifen resistance in breast cancer cells. *ONCOGENE* **26**, 4656-4667 (2007).

2. J. H. Lee, S. M. Jung, K. M. Yang, E. Bae, S. G. Ahn, J. S. Park, D. Seo, M. Kim, J. Ha, J. Lee, J. H. Kim, J. H. Kim, A. Ooshima, J. Park, D. Shin, Y. S. Lee, S. Lee, G. van Loo, J. Jeong, S. J. Kim, S. H. Park, A20 promotes metastasis of aggressive basal-like breast cancers through multi-monoubiquitylation of Snail1. *NAT CELL BIOL* **19**, 1260-1273 (2017).

3. L. Wang, W. Guo, J. Ma, W. Dai, L. Liu, S. Guo, J. Chen, H. Wang, Y. Yang, X. Yi, G. Wang, T. Gao, G. Zhu, C. Li, Aberrant SIRT6 expression contributes to melanoma growth: Role of the autophagy paradox and IGF-AKT signaling. *AUTOPHAGY* **14**, 518-533 (2018).

4. Langan, T. J., Rodgers, K. R. & Chou, R. C. Synchronization of Mammalian Cell Cultures by Serum Deprivation. *Methods Mol Biol*. **1524**, 97-105 (2017).

5. Davidoff, A. N. & Mendelow, B. V. Unexpected cytokinetic effects induced by puromycin include a G2-arrest, a metaphase-mitotic-arrest, and apoptosis. *Leuk Res*. **16**, 1077-1085 (1992).
