# Supplementary figures and images for: A20 promotes melanoma progression via the activation of Akt pathway
Source: Cell Death Dis. 2020 Sep 23;11(9):794. doi: 10.1038/s41419-020-03001-y (PMC7511359; doi:10.1038/s41419-020-03001-y)

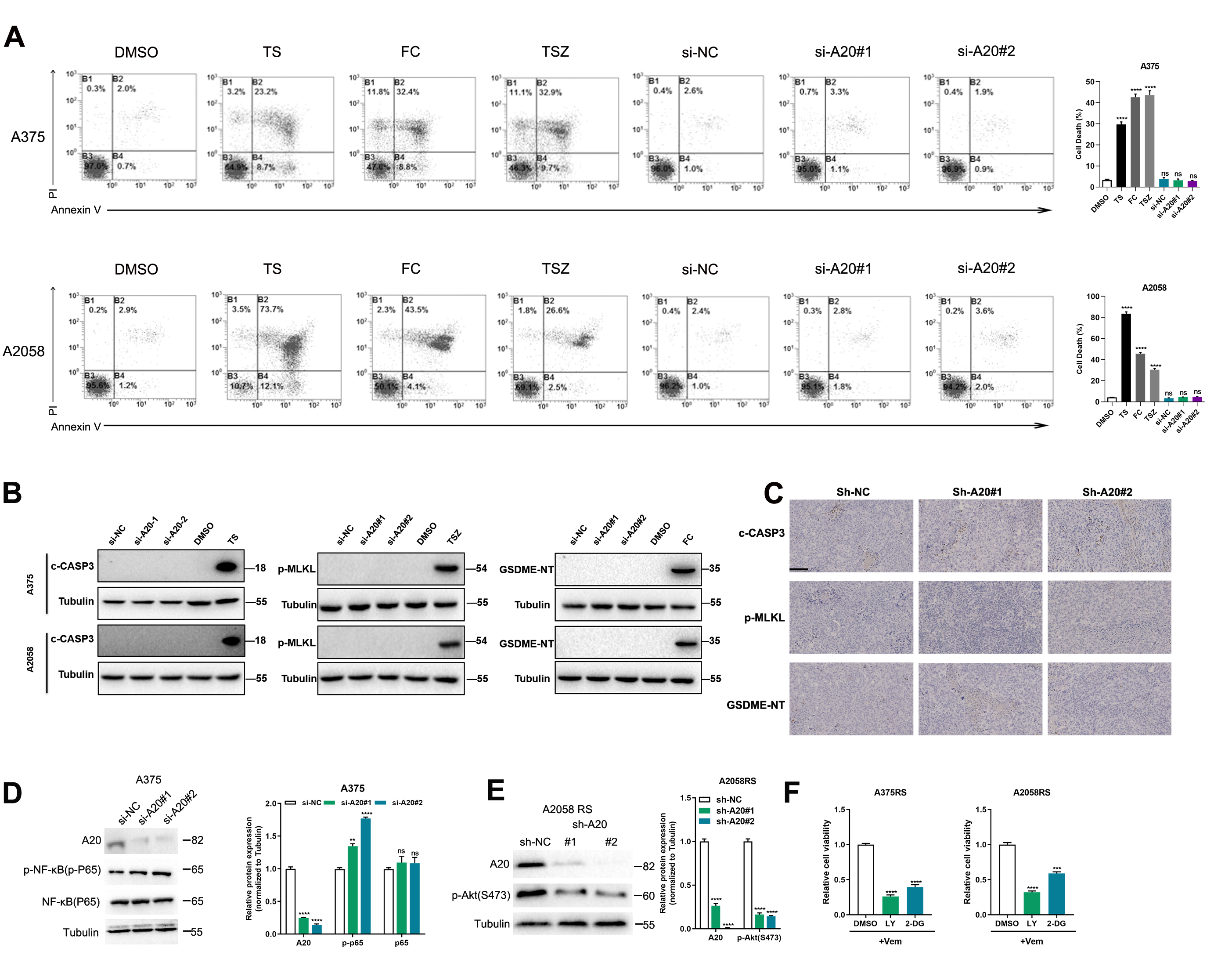

Supplement: Supplementary file 2 — Supplementary Figure S1 [file 41419_2020_3001_MOESM2_ESM.tif]

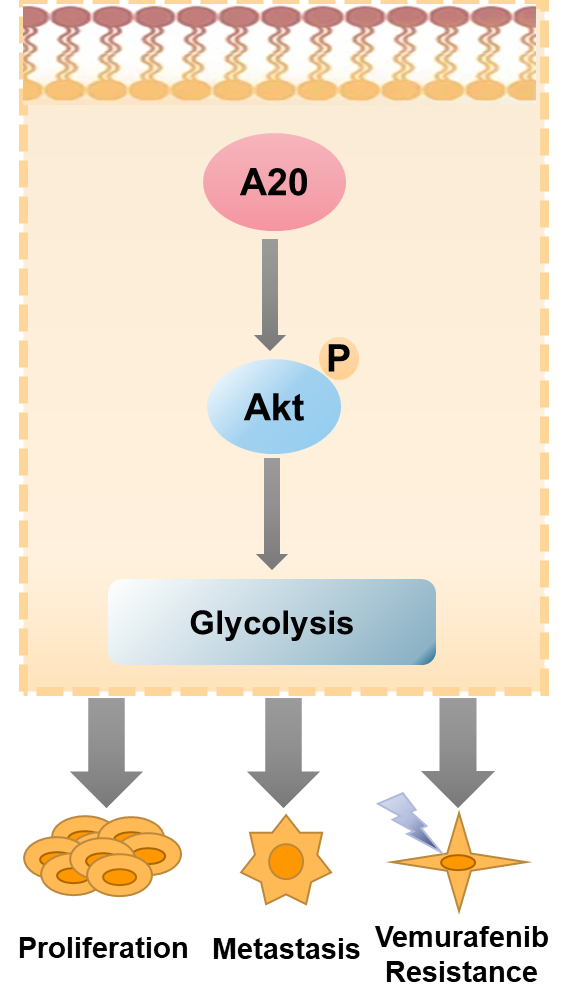

Supplement: Supplementary file 3 — Supplementary Figure S2 [file 41419_2020_3001_MOESM3_ESM.tif]
